# Eight-year chronic wound caused by Tarlov’s cyst: a case report

**DOI:** 10.1186/s13256-023-04232-1

**Published:** 2023-12-07

**Authors:** Ya-Ning Huang, I-Wei Chang, Sung-Tse Li, Wei-Te Lei

**Affiliations:** 1 Department of Pediatrics, Hsinchu Municipal Mackay Children’s Hospital, No.28, Jian Gong 2nd Rd., East Dist., Hsinchu, 300 Taiwan (R.O.C.); 2 Department of Pathology, Taipei Medical University Hospital, Taipei, Taiwan; 3 Department of Medicine, MacKay Medicine College, New Taipei, Taiwan; 4 Department of Healthcare Management, Yuanpei University of Medical Technology, Hsinchu, Taiwan

**Keywords:** Chronic osteomyelitis, Slapping gait pattern, Tethered conus syndrome, Perineural cyst

## Abstract

**Background:**

Tarlov’s cyst is often underdiagnosed since it is difficult to identify without imaging assistance. Herein, we report the case of a young girl who presented with an 8-year history of chronic osteomyelitis of bilateral proximal phalanges and metatarsal bones caused by a Tarlov’s cyst that did not contain a nerve root. The chronic wound in the forefoot is an unusual presentation and resulted from the Tarlov’s cyst accompanied with tethered conus syndrome.

**Case presentation:**

A 10-year-old Asian girl presented with an 8-year history of chronic osteomyelitis of bilateral proximal phalanges and metatarsal bones. She received sequestrectomy five times, however the immune function tests were all normal. A neurological examination revealed diminished sensation and a slapping gait pattern. Magnetic resonance imaging (MRI) demonstrated a lobulated cyst at the right aspect of the sacrum (S) 1 to sacrum (S) 3 canal near the dorsal root ganglion. Tethered conus syndrome was highly suspected. She received laminectomy of lumbar (L) 5 and S1–S2, which led to the diagnosis of a right S1–S3 epidural cyst. The final diagnosis from the histopathological examination was a right sacral Tarlov’s cyst. The clinical conditions of diminished sensation and slapping gait pattern greatly improved after successful surgical treatment.

**Conclusion:**

In children who present with a recalcitrant chronic wound in the forefoot accompanied with a slapping gait pattern and foot hypoesthesia to pain, aggressive imaging examinations such as spine MRI should be arranged for further evaluation, especially in immunocompetent children.

**Supplementary Information:**

The online version contains supplementary material available at 10.1186/s13256-023-04232-1.

## Introduction

Tarlov’s cyst, also known as perineural cyst, is a cerebrospinal fluid-filled sac. It usually forms on the extradural component of sacrococcygeal nerve roots at the junction of dorsal root ganglion and posterior nerve roots [1], and it is often located at the S1 to S5 nerve root area causing corresponding radiculopathy, paresthesia, and muscular weakness. Most Tarlov’s cysts are asymptomatic until they become large enough to cause stretching or exert a compression effect on the nerve root, which is most commonly known as tethered conus syndrome [2, 3]. However, there are currently no reported cases of Tarlov’s cyst associated with a chronic wound for years as in our case. Therefore, the aim of this study was to report the clinical manifestations, imaging study findings, management, and outcomes of this unusual case.

## Case presentation

**Fig. 1:**
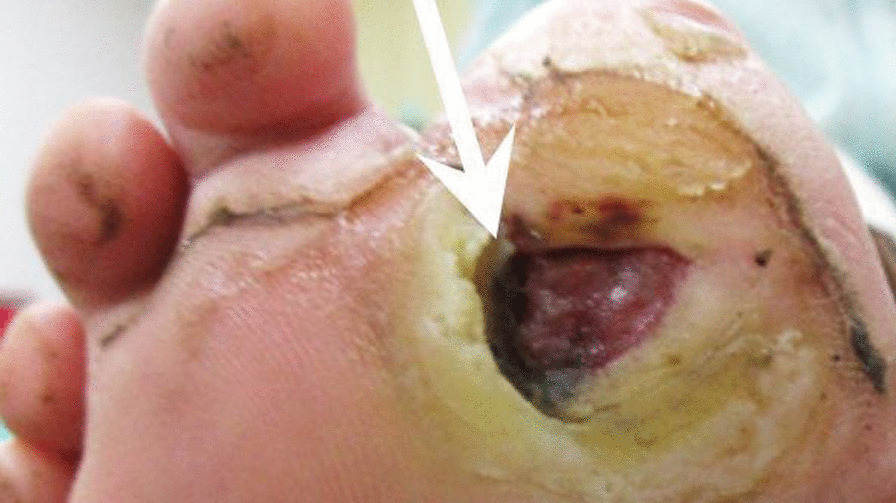
Right forefoot chronic wound 1.5 cm × 0.3 cm in size. Arrowheads pointing the right forefoot wound

**Fig. 2:**
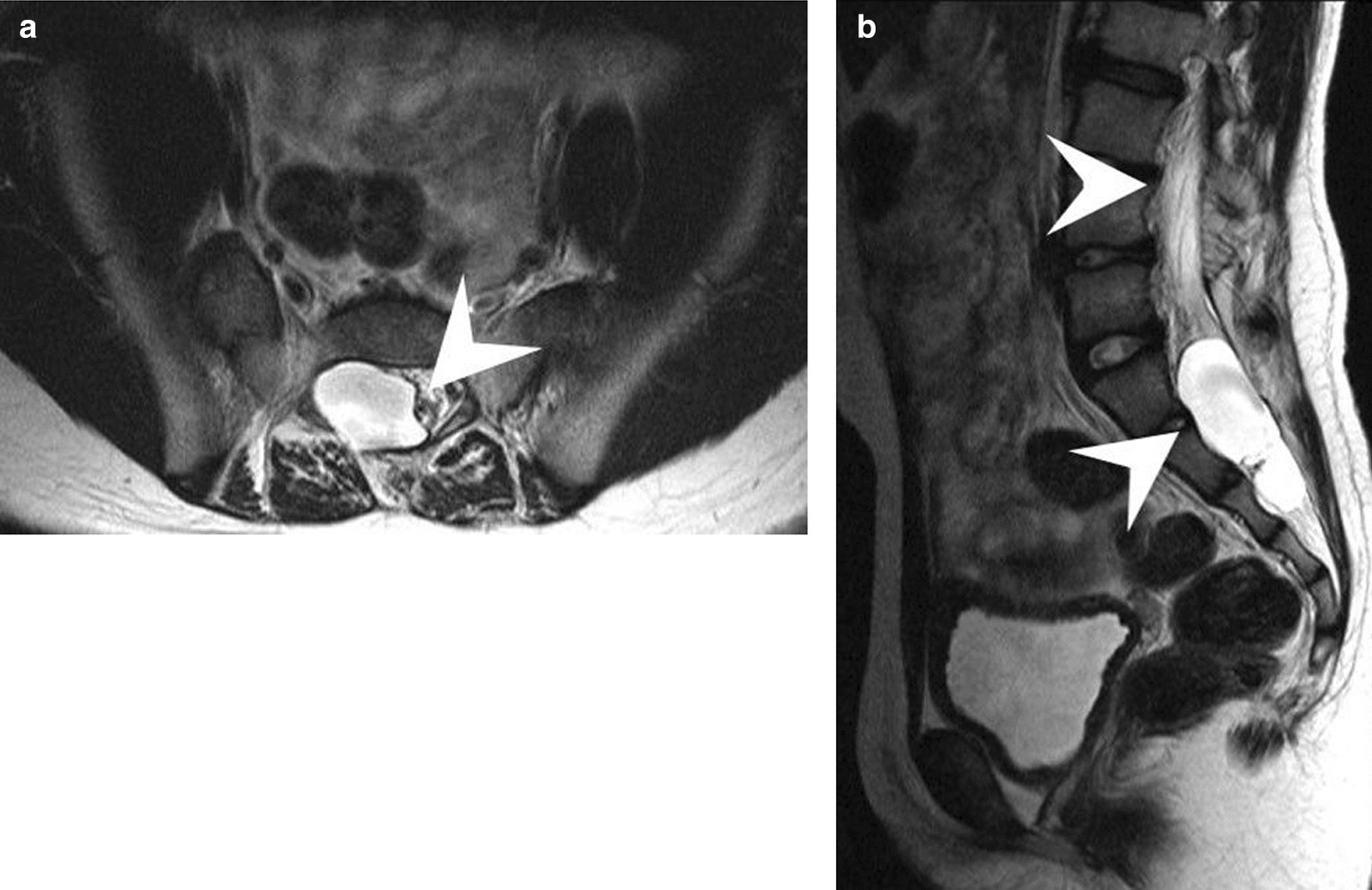
**A** T2 spine MRI demonstrated an S1–3 perineural cyst about 1.8 cm in length and 0.5 cm in diameter with no nerve root within the cyst. Arrowheads pointing the perineural cyst. **B** T2 lumbar spine MRI revealed low lying conus at the L3–L4 interspace with stretching of the filum terminals to the posterior dura wall at the S1–S2 level. upper arrowhead: Low lying conus, lower arrowhead: Posterior dura wall

**Fig. 3:**
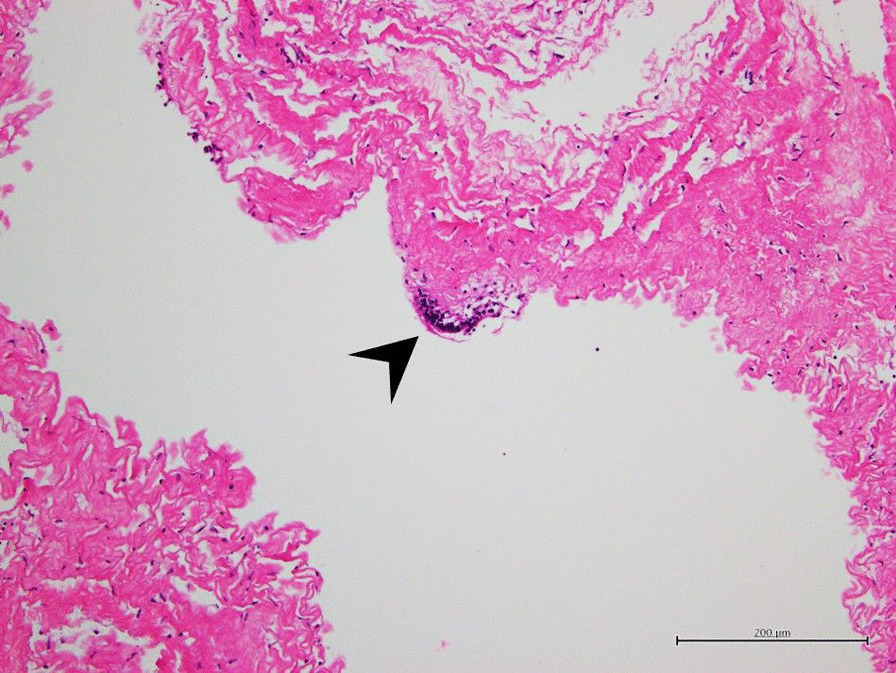
Histopathological examination showed a picture of a fibrous walled cystic lesion with focal meningothelial cell lining as highlighted by EMA immune stain. No nerve fibers were identified. Arrowheads pointing the focal meningothelial cell

A 10-year-old Asian girl presented with an 8-year history of chronic osteomyelitis of bilateral proximal phalanges and metatarsal bones. Since she at young age, she had been prone to fall easily since a young age, and she did not seem to be sensitive to pain. At one and a half years of age, she started to suffer from recurrent cellulitis on both big toes which was refractory to antibiotic treatment, although she was afebrile and had a slightly elevated white cell count and erythrocyte sedimentation rate. She had been gradually able to walk with assistance since she was 8 years of age. Furthermore, she had undergone sequestrectomy five times at 2, 3, 5, 7 and 9 years of age, respectively. As a result, her right big toe had been amputated due to persistent chronic osteomyelitis (Fig. 1). Nevertheless, the immune function tests were all normal. A neurological examination revealed blunt sensations in L4 and L5 dermatomes, especially in bilateral forefoot areas and diminished L4 deep tendon reflex. She also presented with a slapping gait pattern owing to inadequate muscle power of the tibialis anterior which was innervated by a deep peroneal nerve coming from the posterior tibia part of the sciatic nerve in function of L4–L5 [4]. Magnetic resonance imaging (MRI) demonstrated a lobulated cyst at the right aspect of the S1–S3 canal about 1.8 cm × 0.5 cm in size, and no nerve root was contained within the cyst (Fig. 2A). Tethered conus syndrome (Fig. 2B) along with neurogenic bladder was highly suspected. A urodynamic study confirmed the diagnosis of neurogenic bladder. Finally, she received laminectomy of L5 and S1–S2 which led to the diagnosis of a right S1–S3 epidural cyst. The tethering effect of the S1–S3 epidural cyst contributing to the slapping gait was compatible with innervation of the posterior tibia part of the sciatic nerve (L4–L5). The final diagnosis from the histopathological examination was a right S1–3 Tarlov’s cyst (Fig. 3). The clinical condition of diminished sensation and gait pattern greatly improved after successful surgical treatment. This study was approved by the IRB of Mackay Memorial Hospital (18MMHIS208e).

## Discussion and conclusions

Tarlov’s cyst, also known as perineural cyst, is often underdiagnosed since it is difficult to identify without imaging examinations. As a result, most Tarlov’s cysts are asymptomatic until they become large enough to cause stretching or exert a compression effect on the nerve root [2, 3]. There are two types of Tarlov’s cyst, and the type located next to nerve tissue often causes fewer symptoms in early life compared to the type located posterior to the root ganglion with nerve fibers inside. The difference in the course of disease progression is due to the extent of compression on the nerve root [5]. In addition, some studies have reported that neuropathic anesthesia in the lower limbs is caused by spinal cord compression associated with spinal bony defects [6, 7]. A lumbosacral bony defect was also evident in a spine X-ray in our case (Additional files 1 and 2). As in our case, an atypical presentation of Tarlov’s cyst that does not contain a nerve root may delay an accurate diagnosis. Considering the difficulty in making a timely diagnosis, a careful review of imaging studies for symptomatic cysts is crucial.

In children who present with a recalcitrant chronic wound in the forefoot accompanied with a slapping gait pattern and foot hypoesthesia to pain, aggressive imaging examinations such as spine MRI should be arranged for further evaluation, especially in immunocompetent children. The early detection and diagnosis of a symptomatic Tarlov’s cyst with tethered conus syndrome can help to preserve neurologic function and the quality of life.

### Supplementary Information

**Additional file 1.** Thoracolumbar scoliosis. Butterfly vertebra of thoracic(T) 8. Decreased height in T5 to T9 vertebral bodies. Arrowheads pointing the butterfly vertebra of thoracic 8.

**Additional file 2.** Hypoplasia of posterior elements in L3 to L5 vertebrae. Arrowheads pointing the hypoplasia of posterior element.

## Data Availability

Not applicable.

## Abbreviations

MRI: Magnetic resonance imaging
L: Lumbar
S: Sacrum
T: Thoracic

## References

[1] Tarlov IM (1970). Spinal perineurial and meningeal cysts. J Neural Neurosurg Psychiatry.

[2] Feigenbaum F, Henderson F, Benzel E (2012). Tarlov cysts. Spine surgery.

[3] Ju CI, Shin H, Kim SW, Kim HS (2009). Sacral perineural cyst accompanying disc herniation. J Korean Neurosurg Soc.

[4] Ribeiro FS, Bettencourt Pires MA, Silva Junior EX, Casal D, Casanova-Martinez D, Pais D, Goyri-O'Neill JE (2018). Rethinking sciatica in view of a bilateral anatomical variation of the sciatic nerve, with low origin and high division: historical, anatomical and clinical approach. Acta Med Port.

[5] Nabors MW, Pait TG, Byrd EB, Karim NO, Davis DO, Kobrine AI, Rizzoli HV (1988). Updated assessment and current classification of spinal meningeal cysts. J Neurosurg.

[6] Pedro EB, Juan AZ (2008). Painless leg and moving toes syndrome due to spinal cord compression. J Eur Spine.

[7] Tsuyoshi M, Mitsunori Y, Tsuneo T, Toshihiko Y (2010). Case reports: painful limbs/moving extremities: report of two cases. Clin Orthop Relat Res.

