# Supplementary figures and images for: Eight-year chronic wound caused by Tarlov’s cyst: a case report
Source: J Med Case Rep. 2023 Dec 7;17:525. doi: 10.1186/s13256-023-04232-1 (PMC10701988; doi:10.1186/s13256-023-04232-1)

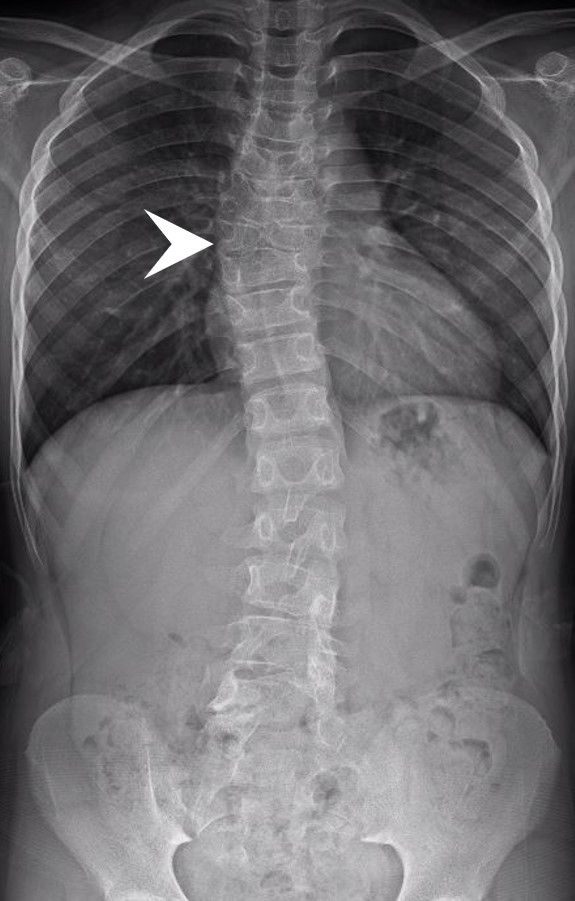

Supplement: Supplementary file 1 — Additional file 1. Thoracolumbar scoliosis. Butterfly vertebra of thoracic(T) 8. Decreased height in T5 to T9 vertebral bodies. Arrowheads pointing the butterfly vertebra of thoracic 8. [file 13256_2023_4232_MOESM1_ESM.jpg]

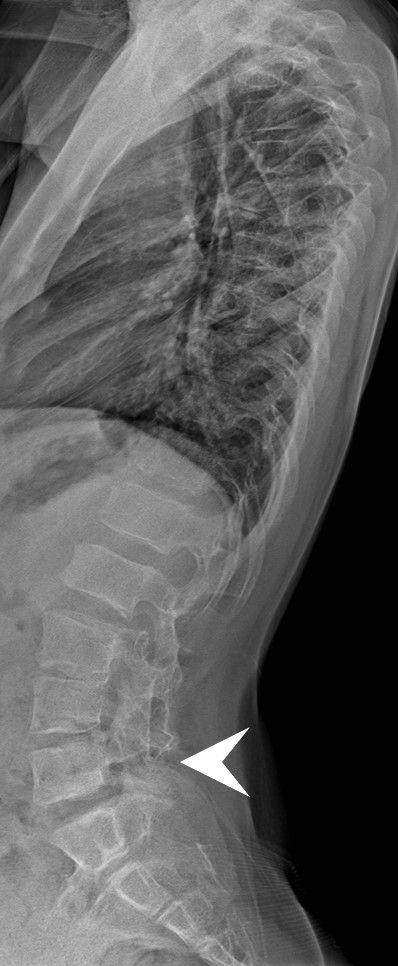

Supplement: Supplementary file 2 — Additional file 2. Hypoplasia of posterior elements in L3 to L5 vertebrae. Arrowheads pointing the hypoplasia of posterior element. [file 13256_2023_4232_MOESM2_ESM.jpg]
